# Economics of Neuraminidase Inhibitor Stockpiling for Pandemic Influenza, Singapore

**DOI:** 10.3201/eid1201.050556

**Published:** 2006-01

**Authors:** Vernon J. Lee, Kai Hong Phua, Mark I. Chen, Angela Chow, Stefan Ma, Kee Tai Goh, Yee Sin Leo

**Affiliations:** * Tan Tock Seng Hospital, Singapore;; † National University of Singapore, Singapore;; ‡ Ministry of Health, Singapore

**Keywords:** Influenza, Treatment, Prophylaxis, Cost-benefit, Cost-effectiveness, Policy, Economic models, Pandemic, research

## Abstract

We compared strategies for stockpiling neuraminidase inhibitors to treat and prevent influenza in Singapore. Cost-benefit and cost-effectiveness analyses, with Monte Carlo simulations, were used to determine economic outcomes. A pandemic in a population of 4.2 million would result in an estimated 525–1,775 deaths, 10,700–38,600 hospitalization days, and economic costs of $0.7 to $2.2 billion Singapore dollars. The treatment-only strategy had optimal economic benefits: stockpiles of antiviral agents for 40% of the population would save an estimated 418 lives and $414 million, at a cost of $52.6 million per shelf-life cycle of the stockpile. Prophylaxis was economically beneficial in high-risk subpopulations, which account for 78% of deaths, and in pandemics in which the death rate was >0.6%. Prophylaxis for pandemics with a 5% case-fatality rate would save 50,000 lives and $81 billion. These models can help policymakers weigh the options for pandemic planning.

## Abstract 2

Stockpiling drugs to prevent and treat influenza would be economically effective.

Ten percent of the world's population and 20% of the population of tropical Singapore are infected with influenza virus annually (*1**,**2*). Amid growing concern about influenza pandemics, national preparedness plans have become essential. In a pandemic hastened by globalization, vaccination is not a viable initial solution because vaccine production requires an estimated 6 months (*1**,**3*). Instead, neuraminidase inhibitors are influenza-specific antiviral agents that figure strongly in preparedness plans. Many nations are acquiring stockpiles of these drugs because of their effectiveness in influenza treatment and prophylaxis (*4*).

Studies have compared the cost-effectiveness of vaccination versus treatment with antiviral agents (*5**–**7*), but only l study has examined the cost-effectiveness of prophylaxis (*8*). We provide further comparison of the economic outcomes of prophylaxis or treatment with antiviral agents to provide national planners with optimal strategies.

## Methods

**Figure 1:**
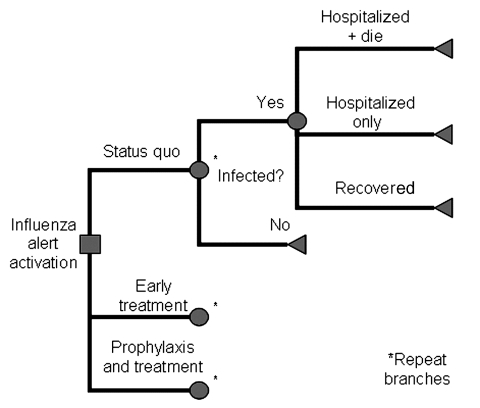
Decision-based model for strategies during pandemic influenza.

This study used a decision-based model (Figure 1) to perform cost-benefit and cost-effectiveness analyses for stockpiling antiviral agents in Singapore. Oseltamivir was the drug of choice because of its safety profile (*9**,**10*) and available data on influenza prophylaxis and treatment (*11**,**12*). The model compared 3 strategies: supportive management (no action), early treatment of clinical influenza with oseltamivir (treatment only), and prophylaxis in addition to early treatment (prophylaxis). Costs were assigned to each outcome, and probabilities at each node were aggregated as population rates for calculating overall costs for each outcome. Decision branches were similar for each strategy, but probabilities at individual nodes differed.

Cost-benefit analyses were used to compare treatment-only and prophylaxis strategies to taking no action. These analyses included direct and indirect economic costs, such as the cost of death. However, quantifying the societal cost of death is difficult, and cost-effectiveness analyses based on cost per life saved by treatment only and prophylaxis, compared to no action, were included. The model was run by using Excel spreadsheets (Microsoft Corp, Redmond, WA, USA); details are shown in the Appendix and on Tan Tock Seng Hospital's website (http://www.ttsh.com.sg/doc/Pandemic%20influenza%20in%20Singapore%20-%20economic%20analysis%20of%20treatment%20and%20prophylaxis%20stockpiling%20strategies.pdf). Costs are represented in 2004 Singapore dollars (2004 exchange rate, USD$1 = SGD$1.6908).

Pandemic influenza is unpredictable: uncertainties surround its occurrence and outcomes (*13*). Excess deaths in annual epidemics occur mostly in the elderly (*14*), but the 1918–1919 Spanish flu pandemic had higher death rates among adults (*15*). To account for such uncertainties, the input variables were modeled as triangular distributions centered on base values, with ranges corresponding to minimum and maximum values (Table 1). Sensitivity analyses, including 1-way analysis, were conducted to identify variables of highest impact and the outcome's sensitivity to treatment and prophylaxis stockpiles. Monte Carlo simulation analyses were performed to determine outcomes under different scenarios.

**Table 1:** Input variables used in analysis*†

Input variables	Age ranges, y			
	<19	20–64	>65	Sources
Average age	10	40	73	16
Population, ×1,000 persons	999.2	2,962.5	278.6	16
	Low risk, %	90	89.7		63.3
	High risk, %‡	10	10.3	36.7	17–20
Baseline influenzalike illness rate, cases/wk	7,686	19,940	750	2,21
Influenza clinical attack rate, % (range)	30 (10–50)	30 (10–50)	30 (10–50)	4,13,22,23
Case-fatality rate/100,000§				Ministry of Health 4,13,24,
	Low risk	5 (1–12.5)	6 (1–9)		340 (28–680)
	High risk	137 (12.6–765)	149 (10–570)		1,700 (276–3,400)
Earnings lost per death, $¶	1,909,092	1,780,027	187,301	16,25
Hospitalization rate/100,000 infected#				Ministry of Health
	Low risk	210 (42–525)	72 (12–108)		1,634 (135–3,268)
	High risk	210 (100–1,173)	234 (16–895)		2,167 (352–4,334)
Average length of hospital stay, d	3.88 (2.3–9.2)	4.61 (3.2–11.8)	6.20 (4.6–13.4)	13,24,26
Average additional days lost	2 (1–3)	2 (1–3)	2 (1–3)	Local physicians
Hospital cost, $/d	342	342	342	Ministry of Health
Value of 1 lost day, $**	108	166/108	108	Ministry of Health, *25*
Outpatient				
	Days lost from outpatient influenza	3 (1–5)	3 (1–5)	3 (1–5)	9,13,23,27
	Consultation and outpatient treatment cost, $	40	40	40	Local physicians
	Value of 1 lost day, $**	108	166	108	Ministry of Health 25,
Treatment with oseltamivir				
	Sought early medical care, %	70 (50–90)	70 (50–90)	70 (50–90)	13,28
	Case-fatality rate reduction, %	70 (50–90)	70 (50–90)	30 (20–90)	24,29
	Hospitalization rate reduction, %	60 (50–90)	60 (50–90)	30 (20–90)	11,24
	Lost days gained, d	1.0 (0.1–2.0)	1.0 (0.1–2.0)	1.0 (0.1–2.0)	7,9,24,28
	Treatment cost, $ per course	31	31	31	Ministry of Health
Prophylaxis with oseltamivir				
	Efficacy of prophylaxis, %	70 (50–90)	70 (50–90)	70 (50–90)	12,30
	Immunity after prophylaxis, %	35 (20–50)	35 (20–50)	35 (20–50)	12,30
	Prophylaxis cost, $/wk	21.7	21.7	21.7	Ministry of Health
	No. stockpile cycles to pandemic	2.25 (1–3.5)	2.25 (1–3.5)	2.25 (1–3.5)	31,32
Pandemic duration, wk	12 (6–24)			32–34
Treatment stockpile, % of population††	10–100			
Prophylaxis stockpile, wk††	2–24			

*All healthcare costs are in 2004 Singapore dollars and were compounded by using the consumer price index for Singapore (*16*). †Base-case values are given with the range used for analysis given in parentheses, where applicable. Input variables were modeled as triangular distributions centered on base values; minimum and maximum values are given by extreme values in ranges. ‡High risk includes asthma, chronic obstructive pulmonary disease, heart disease, and diabetes patients. §Based on deaths among those with clinical influenza. ¶Average present value of future earnings lost per death of a person of average age in the age group. #Rate is based on hospitalizations among those with clinical influenza. Ranges were calculated based on a factor of the base cases versus the death rate. **$166 for lost work day, $108 for unspecified days lost (taking care of ill child or elderly person), and additional days lost after hospitalization. ††The treatment and prophylaxis stockpiles are decision variables, and the analyses were performed for a range of values to determine the preferred outcomes.

Treatment stockpiles, based on proportions of the population, are used on all influenzalike-illness cases, from pandemic plan activation until the pandemic ceases or the stockpile is depleted, whichever comes first. Analysis was conducted to determine the proportion of untreated influenza patients and simulation iterations with complete coverage, by stockpile levels. Further analysis was then performed for prophylaxis stockpiles where prophylaxis, by weeks, is given to the population over and above treatment requirements.

### Input Variables

Input variables are shown in Table 1. Conservative values favoring no action were used to justify alternative strategies. The study was conducted on Singapore's 2004 midyear population of 4,240,300 (*16*), divided into 3 age groups, each consisting of 2 risk groups (low and high risk, according to underlying medical conditions predisposing the patient to influenza complications), for a total of 6 groups that represented differing infection outcomes and drug responses (*13*).

The clinical attack rates during the 1918 and 1957 pandemics were 29.4% and 24%, respectively (*23*), and attack rates in Singapore during the 1967 pandemic were 12.8%–36.4% (*22*). This study assumed a base clinical attack rate of 30% (range 10%–50%), corresponding to rates in other studies (*4**,**13**,**24*).

Case-fatality rates were derived from Singapore's excess deaths from interpandemic influenza; hospitalization and death were assumed to occur only in clinical influenza. To reflect hospitalization rates in relation to case-fatality rates, both rates were correlated. For outpatient visits, clinical influenza patients were assumed to seek medical care and take medical leave. However, some patients may not be treated effectively within 48 hours of infection, and they were assumed not to benefit from treatment.

For pandemic duration, influenza activity in tropical climates commonly rises above the baseline for >12 weeks (*31**,**33*), compared to 6 weeks in temperate climates (*34*). This study assumed a 12-week pandemic duration base value with a range from 6 weeks (average temperate duration) to 24 weeks (assumed vaccine development).

Individual economic value was calculated from the net present value of future earnings for average-aged persons in the respective age groups, adjusted for age. Other costs included were hospitalizations and work days lost; all costs were standardized to 2004 Singapore dollars.

### Oseltamivir

This study relied on international studies on oseltamivir. Oseltamivir has a good safety profile with insignificant rates of severe adverse events and drug withdrawal (*9*). Costs from side effects were thus assumed to be insignificant compared to costs for pandemic illness and deaths. The known safe administration duration of 8 weeks represents only studied durations (*35*). Extension is assumed possible, and the model included up to 24 weeks' prophylaxis. Oseltamivir trials have lacked the power to detect mortality reductions because influenza deaths in trials are rare (*14*), and wide ranges were used to account for uncertainty. Oseltamivir is also less effective in the elderly (*24*). Immunity after prophylaxis among those without clinical infection was assumed to be 35%, as shown during an influenza study in which 38% of study participants on prophylaxis had serologic infection but no clinical infection (*12*). Oseltamivir's pharmacologic action is selective and is assumed to be inactive against noninfluenza illnesses.

Stockpile use depends on the probability of an influenza pandemic occurring. Antigenic shifts and reappearances of past variants were estimated to have pandemic potential every 8–10 years (*31**,**32*). Using oseltamivir's shelf-life of 4 years and patent expiration in 2016, the model assumed a conservative base value of 2.25 stockpile cycles before use (range 1–3.5 cycles) to account for significantly reduced costs after patent expiration. The model assumed that all unused stockpiles are lost.

## Results

If no action were taken during a pandemic, the mean number of simulated deaths in Singapore would be 1,105 (5th and 95th percentiles of 525 and 1,775), with mean hospital days of 23,098 (10,736, 38,638). The mean economic cost would exceed SGD$1.43 billion (0.73, 2.19), and 78% of all deaths would occur in groups at high risk. From the sensitivity analyses, the outcome was most sensitive to changes in attack rate and case-fatality rate reduction with treatment and was sensitive to the variables of treatment and prophylaxis stockpiles.

Table 2 shows the cost and outcomes of various treatment stockpiles; each shelf-like cycle of the stockpile (which is 4 years, after which the drug has to be repurchased) costs SGD$13.1 million for 10% of the population. Stockpiles of <20% did not provide complete coverage in any simulated iterations, while stockpiles of >60% always provided complete coverage. The maximal mean economic benefit of SGD$414 million occurred at a 40% stockpile with 418 lives saved.

**Table 2:** Cost and outcomes with changes in treatment stockpile*†

% stockpile	Cost of stockpile (1 cycle, million $)	Overall % untreated influenza cases	% iterations with complete treatment	Lives saved	Overall benefit over no action (million $)
No action	NA	100	0	Deaths: 1,105 (525, 1,775)	Cost: 1,430 (730, 2,193)
10	13.1	89.1	0	49 (18, 108)	24 (–4, 73)
20	26.3	42.0	0	249 (128, 412)	224 (103, 385)
30	39.4	9.0	15	386 (185, 645)	385 (165, 619)
40	52.6	0.01	55	418 (185, 730)	414 (145, 759)
50	65.7	<0.01	90	422 (185, 744)	399 (122, 761)
60	78.9	0	100	422 (185, 744)	376 (98, 743)
70	92.0	0	100	422 (185, 744)	353 (76, 721)
80	105.2	0	100	422 (185, 744)	330 (52, 700)
90	118.3	0	100	422 (185, 744)	307 (26, 676)
100	131.4	0	100	422 (185, 744)	285 (4, 654)

*Mean values are shown with 5th and 95th percentiles in parentheses; NA, not available. †All healthcare costs are in 2004 Singapore dollars.

**Figure 2:**
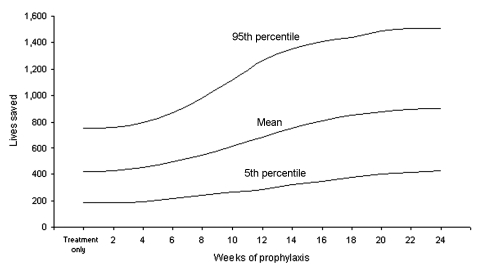
Lives saved compared with no action, by prophylaxis levels. Mean, 5th, and 95th percentiles based on Monte Carlo simulations are shown.

The population cost-benefit and cost-effectiveness outcomes from the Monte Carlo simulation analyses are shown in Table 3. The treatment-only strategy provided the best overall economic benefit, and the no-action strategy was dominated by the treatment-only strategy in cost per life saved.^1^ Each additional week of prophylaxis costs SGD$92 million but reduced the overall economic benefit. Figure 2 shows that increasing the duration of prophylaxis increased lives saved. Lives saved from prophylaxis compared to treatment increased significantly only after prophylaxis of >4 weeks and increased steadily until 20 weeks; costs per life saved also increased.

**Table 3:** Cost-benefit and cost-effectiveness with changes in prophylaxis stockpile for the Singapore population*†

Strategy option	Stockpile cost (1 cycle, million $)	Lives saved compared with no action	Cost per life saved compared with no action ($100,000)	Benefit compared with no action (million $)
No action	Not applicable	Deaths: 1,105 (525, 1,775)	Not applicable	Cost: 1,430 (730, 2,193)
Only Rx‡	79	423 (183, 756)	38 (dominates§, 395)	379 (89, 734)
6 wk¶	631	492 (216, 870)	2,246 (811, 4,676)	–487 (–925, 48)
12 wk¶	1183	684 (286, 1,264)	3,193 (1,008, 6,788)	–1,188 (–1,934, –265)
18 wk¶	1735	850 (377, 1,442)	3,668 (1,358, 7,363)	–1,920 (–2,941, –783)
24 wk¶	2,287	903 (425, 1,509)	4,516 (1,828, 9,022)	–2,811 (–4,070, –1,384)

*Mean values are shown with 5th and 95th percentiles in parentheses. †All healthcare costs are in 2004 Singapore dollars. ‡Only Rx refers to treatment only, without prophylaxis. §Treatment-only dominates no action because treatment-only saves lives and is less costly overall. ¶No. of weeks of prophylaxis for the respective risk and age groups.

Table 4 shows that treatment-only provided the greatest economic benefit across all groups. As prophylaxis duration increased, economic benefit decreased. However, for the 3 groups at high risk (Table 1), the mean overall economic benefit of up to 24 weeks' prophylaxis remained positive compared to that seen if no action was taken.

**Table 4:** Outcomes by age and risk groups*

Risk and age group, y	Strategy option	Stockpile cost (1 cycle, million $)	Mean lives saved compared with no action	Mean cost per life saved compared with no action (million $)	Mean benefit compared with no action (million $)
Low risk, age <1–19	No action	NA	Deaths: 17	NA	Cost: 122
	Only Rx †	17	8	Dominates§	87
	12 wk ‡	251	11	41	–315
	24 wk ‡	485	14	70	–717
Low risk, age 20–64	No action	N/A	Deaths: 42	N/A	Cost: 507
	Only Rx	49	21	Dominates§	382
	12 wk	741	29	40	–808
	24 wk	1,433	36	73	–1,999
Low risk, age >65	No action	NA	Deaths: 185	NA	Cost: 57
	Only Rx	3	60	Dominates§	28
	12 wk	49	108	0.9	–43
	24 wk	95	148	1.3	–115
High risk, age >1–19	No action	NA	Deaths: 92	NA	Cost: 186
	Only Rx	2	45	Dominates§	94
	12 wk	28	63	1.0	83
	24 wk	54	78	1.8	66
High risk, age 20–64	No action	NA	Deaths: 220	NA	Cost: 443
	Only Rx	6	109	Dominates§	235
	12 wk	85	153	1.1	175
	24 wk	165	189	2.0	100
High risk, age >65	No action	NA	Deaths: 547	NA	Cost: 117
	Only Rx	2	179	Dominates§	44
	12 wk	29	321	0.17	24
	24 wk	55	438	0.25	0.1

*Mean values are shown, with all costs in 2004 Singapore dollars; NA, not applicable. †Only Rx refers to treatment-only, without prophylaxis. ‡12 and 24 wk refer to number of weeks of prophylaxis for the respective risk and age groups. §Treatment-only dominates no action because treatment-only saves lives and is less costly overall.

**Figure 3:**
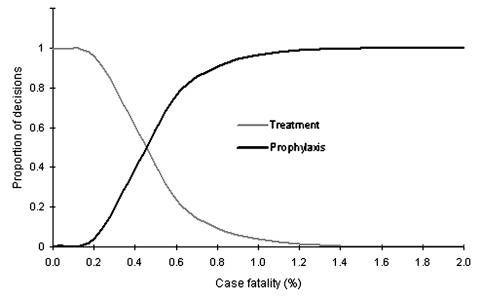
Proportion of decisions for treatment or 24 weeks prophylaxis, by case-fatality rate.

The simulated proportion of decisions with treatment only or 24 weeks' prophylaxis as the optimal outcome is shown in Figure 3. At case-fatality rates of 0.05% (similar to interpandemic epidemics), the decision always favored treatment-only. With increasing case-fatality rates, the decision increasingly favored prophylaxis and intersects between rates of 0.4% and 0.6%. Prophylaxis was always optimal in case-fatality rates of >1.5%. If no action was taken with a 5% case-fatality rate (the 1918 pandemic average) (*23*), 63,000 deaths, 1.5 million hospital days, and economic costs of SGD$112 billion would occur. Treatment-only saved 30,000 lives, benefited the economy by SGD$28–$84 billion, and required 780,000 hospital days. Twenty-four weeks of prophylaxis saved 50,000 lives, benefited the economy by SGD$46–$132 billion, and required 240,000 hospital days.

## Discussion

The analyses suggest that treatment is always beneficial compared to no action and that the optimal treatment stockpile is 40%–60%: 40% maximizes economic benefits, while 60% maximizes treatment benefits. Compared to other strategies, treatment-only was the optimal economic strategy, while no action was always the least desirable option. Although treatment-only saved fewer lives than prophylaxis, stockpiling costs for treatment were lower. Prophylaxis was only economically beneficial compared with no action in subpopulations at high risk.

Substantial outcomes with prophylaxis occurred with durations of >4 weeks because shorter durations prolonged the pandemic, were insufficient for immunity, and did not cover the pandemic's peak. Increasing duration improved outcomes because it covered the pandemic's peak, but the improved outcomes tapered off after 20 weeks, resulting in a sigmoid curve (Figure 2).

In low-risk groups with low death and hospitalization rates, increasing prophylaxis duration decreased economic benefit and increased cost per life saved. In contrast, groups at high risk, who had higher death and hospitalization rates, were affected substantially by prophylaxis, resulting in overall benefits compared to taking no action. Elderly groups had the smallest populations but the highest risk levels and most deaths. However, their lower average future earnings compared to those of younger age groups resulted in lower overall benefits.

This study of pandemic outcomes in a tropical climate is similar to an Israeli study that compared treatment and prophylaxis strategies (*8*). Our study used local health outcome rates but did not include a ring prophylaxis strategy. Both studies found that oseltamivir treatment is economically beneficial, but in addition, our study showed that long-duration prophylaxis is beneficial for high-risk groups and high case-fatality pandemics.

Limitations of this study include the disregard for intangible costs, such as societal value of health; cost-utility analyses could address these costs. Also, indirect effects on national economy and world trade were not considered. For comparability, neither treatment nor prophylaxis was assumed to alter the pandemic's transmission dynamics. This assumption may be true if therapy is limited to small subpopulations, but it understates the benefits if infection is delayed until the pandemic is resolved or vaccine becomes available; it overestimates the benefits if the pandemic continues (*4**,**24*). Correlation between attack rates and pandemic duration was not accounted for, and all possible combinations were included.

### Policy Implications

Stockpiling is insurance in planning for pandemics with high case-fatality rates, in which more severe outcomes and higher risks demand higher premiums. Policymakers should consider lives saved even if economic costs outweigh incremental benefits. Prophylaxis of high-risk groups balances saving lives with economic benefits. Prophylaxis also reduces hospitalizations, which may otherwise overwhelm the healthcare system. Analysis of peak pandemic healthcare use is required to determine the effects of prophylaxis. Other options to reduce a pandemic's impact, including reducing influenza attack rates by quarantine or closing borders, should be considered as alternative strategies.

The current avian influenza (H5N1) outbreak in Asia, which has a high case-fatality rate, indicates the need for decisive action. Oseltamivir is effective against H5N1 and is used as treatment in Vietnam (*36**,**37*). Although resistance has been detected, resistant strains have poor infectivity (*37*). Prophylaxis with oseltamivir will reduce illness, deaths, and economic costs and may reduce spread. If avian influenza develops species crossover with case fatalities exceeding those of the 1918 Spanish influenza pandemic, then stockpiling for treatment and prophylaxis accrues substantial benefits.

The decision to stockpile requires predetermined objectives; noneconomic, moral, and ethical implications should be considered. Treatment-only maximizes economic benefits, while prophylaxis saves most lives. Policymakers have to act decisively, and determine the subpopulations to be given priority, to enable preparedness plans to succeed.

## Appendix

### Details of the Equations Used in the Analysis

Antiviral stockpiles will be used on clinical influenza cases according to the pandemic distribution curve, assumed to be normally distributed (*14*). Baseline influenzalike illness rates are assumed to be constant.

### Proportion Untreated

The population proportion with clinical influenza left untreated because of treatment stockpile deficiencies is calculated as follows:

No. of doses required = (influenzalike illness per week × pandemic duration) + no. of clinical influenza cases

Shortfall of doses for treatment = no. of doses required – no. of doses available

The proportion untreated is the shortfall of treatment doses matched to the number of case-patients who require treatment, according to the pandemic distribution curve.

### Cost of Treatment and Prophylaxis

The cost of treatment was calculated as follows:

Total cost of treatment age_risk group_ = cost of treatment per course × stockpile percentage × population_age, risk group_

The cost of prophylaxis for 1 stockpile cycle was calculated as follows:

Total cost of prophylaxis_age, risk group_ = cost of prophylaxis per week × no. weeks of prophylaxis × population_age, risk group_

### Cost of Outpatient Clinical Influenza

The medical cost of outpatient clinical influenza was calculated as follows:

Outpatient medical costs_age, risk group_ = population_age, risk group_ × attack rate × consultation and treatment cost

The cost of outpatient lost days was calculated by using work days lost for the adult population and unspecified days lost for the young and elderly populations, as follows:

Economic cost of outpatient lost days_age, risk group_ = population_age, risk group_ × attack rate × outpatient days lost × value of a day lost_age, risk group_

### Cost of Hospitalizations

The hospitalization cost for influenza-related complications was calculated by summing direct hospitalization cost with cost of additional days lost after hospitalization.

The direct hospitalization cost was calculated as follows:

Economic cost of hospitalization_age, risk group_ = population_age, risk group_ × attack rate × hospitalization rate _age, risk group_ × length of stay _age, risk group_ × (hospitalization cost + value of a day lost _age, risk group_)

The cost from additional days lost was calculated as follows:

Economic cost of additional days lost after hospitalization = population _age, risk group_ × attack rate × hospitalization rate_age, risk group_ × additional days lost_age, risk group_ × value of a day lost_age, risk group_

### Cost from Influenza Deaths

The cost from influenza deaths is calculated as follows:

Economic cost from influenza deaths = population_age, risk group_ × attack rate × case-fatality rate_age, risk group_ × net present value of future earnings_age, risk group_

### Economic Calculations

For cost-benefit comparisons, the following equation is used:

Overall benefit = overall cost_treatment only or prophylaxis_ – overall cost_no action_

For the cost-effectiveness comparisons, the following equation is used:

Cost per-life-saved compared to no action = (cost excluding cost per life_treatment-only or prophylaxis_ – cost excluding cost per life_no action_) / (deaths_no action_ – deaths_treatment-only or prophylaxis_)

The individual costs that constitute the total costs are calculated for the strategies of no action, treatment-only, and prophylaxis as follows:

Overall cost_no action, treatment-only, prophylaxis_ = Σ (population_age, risk group_ × probability of outcome_clinical influenza, hospitalization, death_ × cost of outcome_clinical influenza, hospitalization, death_ × effectiveness_treatment-only, prophylaxis_) + cost of strategy_treatment-only, prophylaxis_

## Footnotes

1 "Dominate" is a term used in cost-effectiveness analyses and refers to a strategy that is both more efficacious and less costly than another strategy.
